# Supplementary material for: Real-world adolescent smartphone use is associated with improvements in mood: An ecological momentary assessment study
Source: PLoS One. 2024 May 29;19(5):e0298422. doi: 10.1371/journal.pone.0298422 (PMC11135750; doi:10.1371/journal.pone.0298422)
Supplement: Attachment — Submitted filename: Plos_Response_062723.docx [file pone.0298422.s001.docx]

Journal requirements:

When submitting your revision, we need you to address these additional requirements.

1. Please ensure that your manuscript meets PLOS ONE's style requirements, including those for file naming. The PLOS ONE style templates can be found at

https://journals.plos.org/plosone/s/file?id=wjVg/PLOSOne_formatting_sample_main_body.pdf and

<https://journals.plos.org/plosone/s/file?id=ba62/PLOSOne_formatting_sample_title_authors_affiliations.pdf>

Our documents have been edited according to these templates.

2. We noted in your submission details that a portion of your manuscript may have been presented or published elsewhere.

[The data analyzed for this paper were also analyzed for a manuscript that is currently under review at another journal. These analyses were conducted independently, produced different results, and address different theoretical questions --- it is the belief of these authors that each makes a distinct contribution to the scientific literature. As requested, the other manuscript has been uploaded along with this submission.]

Please clarify whether this publication was peer-reviewed and formally published. If this work was previously peer-reviewed and published, in the cover letter please provide the reason that this work does not constitute dual publication and should be included in the current manuscript.

We have added the following language to our cover letter:

“The paper contains original material and is not currently submitted or in press elsewhere in any form. Another manuscript that describes an independent analysis using the same data has been accepted for publication and is currently in press with the journal *Cyberpsychology, Behavior, and Social Networking.* This manuscript, which was submitted with our original publication, describes an analysis that compared the effects of various smartphone apps. This is distinct from the analysis presented in the current manuscript, which examines the relationship between adolescent mood and smartphone use in general.“

3. In your Data Availability statement, you have not specified where the minimal data set underlying the results described in your manuscript can be found. PLOS defines a study's minimal data set as the underlying data used to reach the conclusions drawn in the manuscript and any additional data required to replicate the reported study findings in their entirety. All PLOS journals require that the minimal data set be made fully available. For more information about our data policy, please see http://journals.plos.org/plosone/s/data-availability.

As requested, we have uploaded the data to a publicly-accessible repository and have included information about this repository in our revised Data Availability statement.

4. Please include your full ethics statement in the ‘Methods’ section of your manuscript file. In your statement, please include the full name of the IRB or ethics committee who approved or waived your study, as well as whether or not you obtained informed written or verbal consent. If consent was waived for your study, please include this information in your statement as well.

As requested, we have added our Ethics statement to the ‘Methods’ section of our manuscript.

5. Please include a separate caption for each figure in your manuscript.

We have uploaded our captions as a supporting information file as per instructions.

6. Please review your reference list to ensure that it is complete and correct. If you have cited papers that have been retracted, please include the rationale for doing so in the manuscript text, or remove these references and replace them with relevant current references. Any changes to the reference list should be mentioned in the rebuttal letter that accompanies your revised manuscript. If you need to cite a retracted article, indicate the article’s retracted status in the References list and also include a citation and full reference for the retraction notice.

Our reference list has been reviewed as requested.

1. For a better detailing into the introduction as a contribution, relevance, and theoretical cut, the opening could be organized using paragraphs as follows:

We appreciate the suggestion to adopt this structure, which we feel has greatly improved the introduction to the manuscript. The paragraphs of our restructured introduction are presented below.

(i) Common ground (what we know in the literature): The author must present the basic premises of the literature under analysis to establish a starting point for the reader.

In the 15 years since the iPhone and Android OS were introduced to the market, smartphone use has become nearly ubiquitous among U.S. adolescents—about 95% now report having access to a personal smartphone and 46% say they are connected to the internet “almost constantly”[1]. Rates of adolescent depression[2,3], self-harm[4], and suicide[5] have also risen over this period, leading some to suggest that smartphone use is detrimental to adolescents’ emotional well-being[6]. This fear is widely shared among parents[7], and some scholars have called for policies to regulate or restrict adolescent use of smartphones and social media[8,9].

There is evidence that some specific patterns of digital technology use are associated with adolescent mood problems. For example, adolescents who exhibit problematic or compulsive smartphone use are more likely to be depressed[10], experience more mood disturbances[11], and have less-developed emotion regulation skills[12]. Recent reviews have also associated symptoms of depression with adolescent social media use[10,13], but authors of these studies noted that effects vary greatly across studies and might be related to some patterns of social media use but not others. Indeed, studies that distinguish between passive (i.e. scrolling through social media feeds without posting or interacting) and active patterns of social media use generally find that depressed moods are more strongly associated with passive use[14]. Thus, there is evidence that the moods and emotional health of adolescents are negatively associated with *specific types* of smartphone use: problematic use and passive social media use. **(page 4)**

(ii) Complication (what are the limitations – gaps – of the literature): The author must expose missing elements or flaws in the literature (incoherent, incomplete, or contradictory theories) to explain some phenomenon that it should explain. Practical, real-world examples can help expose the limitations of the literature.

Research into the broad effects of smartphone use (i.e. effects that are not constrained to a specific application or pattern of use) has so far provided mixed and often contradictory conclusions. Some scholars have directly associated the recent rise in mood problems with increases in technology use[6,9,15], but others report that technology use has positive[16] or neutral[17] effects. Some recent reviews and meta-analyses have observed small negative effects of smartphone use on adolescent mental health[18,19] while others suggest effects are mixed and uncertain[20,21]. Adolescents themselves are also divided on the issue—24% of respondents to a recent poll said digital media use had “mostly negative” effects on their lives while 31% reported “mostly positive” effects[22]. Thus, it remains unclear whether smartphone use in general has any effects on adolescents’ moods or mental health. This may be due to methodological limitations in the existing literature [15], three of which are reviewed below.

# Overreliance on recalled estimates of smartphone use

Research into the effects of technology use of all kinds—from smartphone use to social media use to overall screen time—often measures quantity or frequency of use through one-time, self-reported recalled estimates. For example, past studies have asked participants to estimate the total number of minutes they spend on their phone each day[23] or week[24], or to estimate how many times they pick up their phones during the day[25]. A recent systematic review and meta-analysis found that measures like these have limited validity because they are only moderately correlated with more objective measures of screen time use[26]. Further, people with lower psychological well-being have been found to overestimate their smartphone and social media use when asked to offer recalled estimates, falsely inflating the associations between smartphone use and mental health concerns[24]. These problems may be exacerbated among adolescents, who overestimate the amount of time they spend on social media[27].

Recalled estimates are often used to measure smartphone use in surveys, which are the most common data collection techniques used in this area of research[28,15]. Meta-analyses and systematic reviews that have found associations between technology use and mood[18,21] have drawn primarily from studies that used recalled estimates, so it is possible that their findings were affected by participants’ recall bias. Recognizing this as a serious gap in the literature, scholars have recently called for the use of measures that are less sensitive to the biases associated to estimation and recall[29]. One possible approach is the use of *in-situ* measures of smartphone use like those facilitated by Ecological Momentary Assessment (EMA) methods. These measures capture smartphone use in the moments it occurs, which facilitates easier estimations and greatly reduces the recall bias[30]. Indeed, research has found that *in-situ* measures of adolescent social media use delivered much smaller estimates of smartphone use than self-reported recalled estimates[31]. Thus, the current understanding of the interaction between smartphone use and adolescent mood is largely built around measures of smartphone use that are known to be biased.

# Failure to observe short-term, within-participant associations

Another reason that scholars struggle to find consistent associations between adolescent smartphone use and mood may be that these associations exist at a within-participants level on a timescale that cross-sectional data cannot capture. A recent review of research into the effects of digital technology use on adolescent well-being suggested that technology use is more likely to have transient short-term effects on adolescents’ emotions than on long-term emotional health outcomes[15]. Such effects would be mostly invisible to studies of cross-sectional data, which are fixed at a single moment in time. Scholars have thus called for more experimental and longitudinal research[14,15,29].

The limited experimental and longitudinal research performed so far suggests that digital technology use can indeed have short-term effects on emotional states, but the strength and valence of observed effects varies depending on the type of technology use. For example, experiences of acceptance on social media (such as receiving a “like”) tend to increase activity in brain regions associated with reward[32,33] while rejection experiences interact with networks associated with depression and negative affect[34,35]. Further, experimenters who induced passive social media use found it immediately increased boredom[36]. In longitudinal studies, passive social media use has been found to precede and predict depressed mood among adolescents[37], while intentional management of a Facebook profile was found to predict improvements in self-esteem[38]. Notably, these studies manipulated the use of specific smartphone apps or styles of smartphone use---little is known about the within-participant effects of smartphone use in general.

# Lack of evidence for temporal precedence

Cross-sectional designs are also limited because they do not allow researchers to determine whether smartphone use precedes or follows its supposed effects[29]. Temporal precedence is considered essential to causation—if it cannot be established, any observed relationships must instead be considered correlational. If smartphone use is found to have short-term, within-participant associations with mood, this might be explained in two ways. Smartphone use could cause changes in mood (e.g. using a smartphone causes adolescents to feel depressed), or it could be that moods drive adolescents to use their smartphones (e.g. adolescents are more likely to use their phones when they feel depressed). **(pages 5-7)**

(iii) Concern: The author must show that the limitation of the literature is something relevant to be studied. More than a trivial limitation is required to motivate a search. The author should write about How and Why restriction impairs our understanding of a phenomenon.

In sum, inconsistent findings about the relationship between smartphone use and adolescent mood may be attributable to methodological limitations. Much research in this area has relied on data collected through cross-sectional surveys, which often use biased measures, are insensitive to within-participant effects, and cannot be used to establish causality. Experimental data in this field is also limited because it has generally focused on specific patterns of smartphone use and because it is usually conducted in a controlled environment. These approaches are unable to detect the transient, moment-to-moment effects of real-world smartphone use on mood, which may be more reliable than associations with longer-term outcomes like life satisfaction and well-being [15]. Thus, the current literature is not able to verify or refute claims that smartphone use might be harmful to adolescents’ mental or emotional health. **(page 8)**

(iv) Course of Action: The author should show the course of action that may resolve the central complication. To resolve the limitation, the author must develop a theory or refine an existing theory. To this end, the author can (i) suggest new constructs; (ii) model new relationships between constructs; (iii) explore the theoretical process; (iv) develop a typology. (v) Contribution: The author must explain how his course of action can contribute significantly to the existing theory (that which has been described on the common ground).

The present study sought to address these gaps by using an Ecological Momentary Assessment (EMA) data collection protocol, which allowed for *in-situ* measures of both adolescent smartphone use and mood. This approach allowed the study team to capture the interactions between these variables in real time in a real-world setting and afforded the ability to examine within-participant associations while controlling for differences between participants. Further, this protocol allowed researchers to compare adolescent mood during smartphone use with moods before smartphone use, allowing for inference about temporal orientation**. (page 8)**

2. The authors could address a formal objective at the end of the introduction; in this sense, the reader will understand better the course perspective.

We have added the following language to the end of our introduction:

**Study objective**

The goal of this study was to test whether real-world smartphone use might have reliable but transient effects on adolescents’ moods, using an *in-situ* Ecological Momentary Assessment approach.

*RQ1: Does adolescent smartphone use have short-term, within-participant associations with mood?*

*RQ2: Do adolescents report changes in mood after starting smartphone use?* **(pages 8-9)**

3. The limitations presented could be discussed in a longitudinal perspective aligned with state of the art about the central construct: the mood in a specific context, an adolescent.

We appreciate this insight and agree that the current paper is limited because it does not consider how the relationship between adolescents’ smartphone use and mood might change over time. We have added the following language acknowledging this limitation to our “Limitations and recommendations for future research” section:

Fourth, the analysis used for this study was limited because it did not consider how effects of smartphone use on mood might vary over time. Time is known to be an important consideration of media effects because these effects are sometimes delayed [55] or build gradually over repeated exposures [56]. The present study was concerned with the possibility of transient, in-the-moment associations between smartphone use and mood, but we suggest that future work investigate the possibility of more complex longitudinal relationships. Specifically, we advise the use of multilevel cross-lagged panel models, which have been used previously to effectively identify patterns in the appearance and duration of media effects [57]. **(page 21)**

4. The research questions appear on page 8 and could be addressed with the limitations and theory discussion.

We have edited the opening sentences of our Discussion section to re-state our study objective and research questions:

The goal of this study was to test whether real-world smartphone use might have reliable but transient effects on adolescents’ moods, using an *in-situ* Ecological Momentary Assessment approach. Toward that end, we explored two research questions. First, we investigated whether adolescent smartphone use had short-term, within-participant associations with mood. Second, we tested whether adolescents reported changes in mood after starting smartphone use. **(pages 16-17)**

5. the period the authors choose to collect data needs to be clarified.

We now describe the dates in which data was collected as follows:

The study was conducted in five independent waves between October of 2019 and March of 2020 **(page 9)**

6. Excellent perspective in choosing longitudinal and multilevel modeling procedures.

Thank you!

7. For better replicability of the study, the authors could present a flowchart of the analytical proposal.

We have included with this new submission a general illustration of the models tested in this study (Model_RQ1.pdf & Model_RQ2.pdf). We make these available to the editor as resources that will be provided by the corresponding author in response to any requests.

8. When presenting the experimental perspective, it would be interesting to discuss the modeling of the levels of the variables and if the proposed scales were updated based on the literature through validation.

To better clarify our model, we have added language to our Methods section describing the level (participant-level or assessment-level) of each variable.

9. Could approach the analysis from the gender of the respondent for a comparison of different perspectives and effects.

Past literature suggests that the effects observed in this study may vary as a factor of many individual differences, including not only gender but also age and race. For this reason, we feel it would raise questions for readers if analyses were presented for one individual difference but not others. We agree that these differences present intriguing opportunities for future research, however, and have added language to that effect to our “Limitations and recommendations for future research:

Third, future work should explore the possibility that the associations observed in this study might differ as a factor of stable individual differences that were not modeled in our analyses. For example, research has shown that adolescent girls have different motivations for smartphone use than adolescent boys [53], and adolescents’ propensity to develop harmful or problematic smartphone use is related to socioeconomic status, parental relationships, and other factors [41]. Further, it known that media effects generally tend to vary as a factor of individual differences [54] By exploring the ways that moment-to-moment effects of smartphone use differ across different demographic groups, future studies may uncover reasons that some adolescents develop problematic patterns of smartphone use while others do not. **(page 20)**
