# Supplementary material for: Real-world adolescent smartphone use is associated with improvements in mood: An ecological momentary assessment study
Source: PLoS One. 2024 May 29;19(5):e0298422. doi: 10.1371/journal.pone.0298422 (PMC11135750; doi:10.1371/journal.pone.0298422)
Supplement: Attachment — Submitted filename: Response to Reviewers.docx [file pone.0298422.s002.docx]

We appreciate the thoughtful comments of both reviewers, which we feel have substantially improved the manuscript. As the two reviewers have identified many similar issues, we have grouped our responses together below:

In adapting our analyses to accommodate reviewer feedback, we also excluded eight participants from our analysis due to missing data. We have thus updated the description of our data cleaning procedure (on p. 10), and descriptive statistics throughout the manuscript have been updated to reflect the removal of these participants. Removal of these participants did not meaningfully affect the results of either regression analysis.

Regarding the availability of data, all data used in this study are available to through the [TAM Data Consortium](https://tamprogram.org/tam-data-consortium/).

**Item one: Risk of self-report bias**

R1: “Measures of phone use and time on the phone were self-reported. The authors acknowledge this as a limitation. Although the immediacy of the assessment obviates any significant recall bias, other self-report bias may be present.”

R2: “Regarding the methods, the authors indicate that both the phone use and the time spent were reported by the users themselves, a limitation recognized due to the possibility of self-report biases, despite the reduction of memory biases due to the immediacy of data collection.”

We have expanded our description of this limitation to include the possibility of additional self-report biases:

“Importantly, these reports may also be vulnerable to other biases associated with self-report [54]. For example, some participants may have underreported *length of phone use* due to social desirability pressures.”(p.20-21)

**Item two: Risk of recall bias**

R1: “Mood before smartphone use was self-reported when the EMA captured an episode of smart phone use. This could be seen as problematic. An alternative approach could be to use mood reported at the previous EMA, although this might not capture very short-term effects.”

R2: “The mood before smartphone use was also reported by the users at the moment the Event-Contingent Self-Monitoring (EMA) detected device use. This method could be problematic, and an alternative suggested would be to employ the mood reported at the previous EMA to capture mood variations, although this might overlook very rapid fluctuations.”

We appreciate this insight and agree that the recalled nature of our *mood before smartphone use* is an important limitation of this study. As the reviewer has noted, we feel that comparison of *current mood* ratings with ratings given in previous assessments is equally problematic because the timing of assessments was too sparse to capture the very short-term effects this study was interested in.

**Item three: Description of random effects**

R1: “Authors state: ‘To account for the possibility that some individuals used their phones more often than others, we modeled a random intercept for using smartphone.’ and ‘We also modeled a random intercept and slope for length of smartphone use, for similar reasons’ This is unclear, as intercepts are not attached to the independent variable. I assume the authors mean that they included a random subject intercept (which accounts for intra-person correlation of mood observations), and random slopes for smart phone use and length of smartphone use. Perhaps the authors could explain how including a random intercept accounts for variation in frequency of phone use.”

R2: “The authors mention that they modeled a random intercept to account for individual differences in the frequency of smartphone use, as well as a random intercept and slope for the duration of use, which may have been confusing due to a lack of clarity on how these intercepts relate to the independent variable. They could have offered a more detailed explanation of how this modeling helps to consider variations in frequency and duration of use among individuals.”

Thank you for this note, which highlights a flaw in the description of our methodology and provides an opportunity for additional clarity. We have edited the description of our model as follows:

“The model included by-participant random intercepts to account for intra-participant correlations in mood ratings, and by-participant random slopes for both *using smartphone* and *length of smartphone use* to account for participant level differences in the strength of those effects.” (p. 13)

**Item four: Choice of analysis for RQ2**

R1: “On the analysis for RQ2, authors state “we conducted a two-tailed single-sample t-test on change in mood scores to determine whether they were significantly positive or negative.” Again this is unclear. I must assume the authors mean that they conducted a paired t-test on mood reported at EMA vs. reported mood prior to phone use. This would not adjust for intra-person correlation of repeated mood observations.”

R2: “Furthermore, the analysis for the second research question apparently involved a two-tailed single-sample t-test, though the description is ambiguous. It is presumed that the authors applied a paired t-test, comparing the mood reported at the EMA with the mood reported previously, a technique that does not account for the intra-personal correlation of repeated mood observations.”

We appreciate this comment, which identified a way in which our analysis could be improved. In response, we have adjusted the analysis for our second research question. We now use multilevel modeling to assess the effects of phone use on changes in mood while controlling for intra-participant correlations:

“We also adjusted the structure of the data so that both *current mood* and *mood before smartphone use* were denoted as *mood*, with a separate *time* variable noting whether the mood was reported before or during smartphone use. We tested the fit of a linear mixed effects model in which *mood* served as the outcome variable and *time of day,* *weekend, age*, *gender*, *race, ethnicity, free lunch, depression, time,* and *length of smartphone use* served as predictors. This model included a by-participant random intercept to account for intra-participant correlations in mood, and by-participant random slopes for the effects of *time* and *length of smartphone use*.” (p. 14)

**Item five: Inclusion of time of day and day of week effects**

R1: “The analysis did not include day of week or time of day effects.”

R2: “Interestingly, the analysis did not consider the effects of the day of the week or the time of day…”

We agree that this is an important limitation of our analysis, and so we have added *time of day* (hour) and *day of week* (weekday/weekend) as fixed effects in both of our multilevel models. We have also added a section explaining these predictors:

“Past research suggests that adolescent moods exhibit certain predictable patterns over time, with adolescents reporting mood improvement over the course of the day [41] and more positive affect on weekend days [42]. Thus, we used the timestamp associated with each assessment to denote the *time of day* and *day of week* in which the assessment was completed. Because past work notes an important distinction between weekdays and weekends, we categorized *day of week* into weekday (Monday – Friday) and weekend (Saturday – Sunday) days.” (p. 11)

**Item six: Clarification of analysis software**

R1: “Software used for analysis was not specified”

R2: “…the authors did not specify the software used for the analysis. This might impact the interpretation of the results and the replicability of the study.”

This has been clarified with the following language:

“Analyses were conducted in R version 4.3.2 (2023-10-31) [42] using the lme4 package [43]” (p. 13

**Item seven: Presentation of model results**

R1: “It would be helpful to include tables of the model results, including random effects parameters.”

R2: “In the results, it would be beneficial to include tables detailing the model findings, especially the parameters of the random effects.”

We have now added such a table for each of the two multilevel regression models. =

**Item eight: Clarification about figure 1**

R1: “On figure 1, is this based on raw scores or marginal predictions?”

R2: “There's also a question about whether Figure 1 represents raw scores or marginal predictions, a detail that could clarify the interpretation of the data.”

We have added language to the Figure 1 caption clarifying that this figure was created using raw scores. This new language can be found in the supplemental file S2_FigCaption_edited.docx.

**Item nine: Discussion of possible confounders**

R1: “The authors do not discuss possible unmeasured confounders that may explain the correlation between phone use and mood.”

R2: “In the discussion, the authors omitted a consideration of possible unmeasured confounding variables that could influence the observed correlation between phone use and mood”

We have added the following language to our limitation section urging future attention to moment-by-moment confounders:

“Similarly, we recommend that future studies attend more to the possibility of moment-to-moment confounders. For example, it is possible that participants in our study reported higher moods while using their phones partly because phone use coincided with periods of leisure time.” (p. 22)
